# Personalizing Digital Health: Adapting Health Technology Systems to Meet the Needs of Different Older Populations

**DOI:** 10.3390/healthcare11152140

**Published:** 2023-07-26

**Authors:** Jennifer Jimenez, Alberto del Rio, Arianna N. Berman, Miriam Grande

**Affiliations:** 1 Asociación Parkinson Madrid, 28014 Madrid, Spain; arianna.berman@tufts.edu (A.N.B.); miriamgrande@parkinsonmadrid.org (M.G.); 2 GATV Research Group, Signals, Systems and Radiocommunications Department, Universidad Politécnica de Madrid, 28040 Madrid, Spain; arp@gatv.ssr.upm.es

**Keywords:** ageing population, chronic illness, digital healthcare personalization, healthcare systems, healthcare system evaluation

## Abstract

The ageing of the population is growing significantly and will challenge healthcare systems. Chronic diseases in the older population require a change in service delivery, and new technologies can be a key element in ensuring the viability and sustainability of these systems. However, the generation gap and the physical and cognitive decline commonly associated with the older generation are barriers to the transition to these models of care. Despite this, there has been a trend towards digital healthcare, which has many potential benefits for the older population. Numerous studies have assessed the acceptability of new technologies for older people in healthcare. These studies highlight the importance of perceived usefulness, compatibility, ease of use and personalisation of the technology. Personalisation is necessary to ensure that the system is useful for users, and different characteristics such as country of origin, gender, age, or comfort with the technology should be taken into account. A person-centred approach in the development of new health technology systems is essential to ensure that applications can be better tailored to the needs of different ageing populations. Many organisations have dedicated time and resources to ensure a person-centred approach in the development of new health technology systems, and putting the individual first is the best way forward in digital health. This article presents the work carried out in this regard in the framework of the European TeNDER project together with an analysis of the results obtained in terms of satisfaction, usefulness, and usability from end-users. The dynamic and continuous process carried out throughout the TeNDER project translates the needs reported by users, as far as personalisation of interactions is concerned. All end-users held a positive opinion about the usability and usefulness of the system.

## 1. Introduction

In recent years, different organisations have dedicated time and resources to ensure a person-centred approach in the development of new health technology systems. In 2020, the WHO published a proposal regarding digital technologies and global health that highlights its commitment to support human-centred rather than disease-centred technologies [1]. Additionally, many countries have undergone projects in Digital Health Strategies with the common goal of personalising health technology systems [2,3,4,5,6,7]. The Smart Health Systems International comparison of digital strategies of 17 countries (Australia, Belgium, Denmark, Estonia, France, Germany, Israel, Italy, Canada, The United Kingdom, The Netherlands, Austria, Poland, Portugal, Sweden, Switzerland, and Spain) produced some key findings [8]. This research demonstrated that (a) prioritising the individual is the best way to progress digital health, (b) it is vital that health technology systems adapt to the varying needs of each end-user, and (c) hesitancy and resistance to changes resulting from the digitisation can be expected.

The exploration of Digital Health Strategies implemented by various countries highlights the growing significance of leveraging technology to improve healthcare for older individuals. It becomes evident that adopting a person-centred approach in the development of digital health systems holds substantial promise in better tailoring applications to meet the diverse needs of older populations. By focusing on personalized solutions, we can maximize the potential benefits that digital health offers to older individuals and enhance their overall well-being.

The TeNDER system is a tool designed to support individuals affected by chronic diseases such as Alzheimer’s Disease (AD), Parkinson’s Disease (PD), and cardiovascular diseases (CVD) [9]. These conditions necessitate long-term care and continuous medical attention, emphasizing the importance of maintaining healthy habits in daily life. The system addresses this need by empowering patients through a sensorial ecosystem, enabling them to stay informed about their health, adhere to treatment, and preserve their autonomy and quality of life [10]. It was implemented and tested in several countries in Europe (Spain, Italy, Slovenia, and Germany), with tests involving several types of participants (patients, carers, and socio-healthcare professionals). This integrated care system is defined as a set of actions aimed at realising a new organisation of care in order to assist, support, and care for those who need it, as well as to recognise, reduce, and redistribute care work. The system consists of a web app (for professionals), a mobile app (for patients and caregivers), and a series of sensors and devices (wristband, binary sensors, depth cameras, among others) thanks to which different symptoms are monitored.

In addition to the technical modules, the TeNDER system incorporates a human workforce to provide comprehensive support for patients. Within the project, professionals play a crucial role, actively participating in the care for and attention to patients while providing valuable assistance to caregivers. These professionals encompass a diverse range of disciplines, including physicians, nurses, physiotherapists, social workers, and more. Their direct involvement in the care process ensures a multidisciplinary approach and enhances the overall quality of care delivered to patients within the TeNDER system.

A previous study [11] explored the use of wearables in the elderly to promote active self-care. They concluded that the social aspect was the most important aspect for the use of wearables, focusing on family and close contacts as the main drivers for convincing patients. They also concluded that another aspect of willingness to use wearables was easy access and better connectivity to the healthcare system but did not explore this further. The TeNDER system directly addresses these points, as it facilitates seamless interaction between patients, caregivers, and healthcare professionals, fostering continuous awareness and real-time updates on patients’ health status [12,13].

Personalizing the development and implementation of the TeNDER system lies at the heart of its mission. The co-design process employed to create the TeNDER system places users at the forefront and incorporates their specific needs into every component. By tailoring the system to address the unique requirements of individual patients and clinicians, while also ensuring accessibility for those facing technological barriers, the TeNDER system becomes a vital bridge between users and assistance systems. Achieving this level of personalization necessitates utilizing high-quality data and continually evolving the system through various pilots. By increasing the degree of personalization in interactions, the TeNDER system enhances user confidence and maximizes the benefits derived from their engagement with the project and its derived tools.

Population studies predict that the European population of older people (defined as those aged 65 and over) will increase significantly from 90.5 million at the beginning of 2019 to 129.8 million in 2050. Their relative share of the total population will also gradually increase and is projected to reach 29.4% in 2050. During this period, the number of people aged 75–84 in the EU-27 is projected to increase by 60.5%, while the number of people aged 65–74 will increase by 17.6% [14].

Given these figures, it is easy to predict the major impact that these demographic changes will have on the health and care needs of the Member States. A population with a higher percentage of older people will mean a greater number of chronically ill people to be cared for by European healthcare systems, making it necessary to change the provision of services, and in this area, new technologies can be a key element in guaranteeing the viability and sustainability of these systems.

It is important to underline that despite the potential of new technologies to support care for the chronically ill, the generation gap [15,16] is a major obstacle to the transition to these models of care, which is why actions are needed to help reduce it and facilitate the acceptance and usability of the systems [15,16]. Elderly individuals struggle more with technology [17,18] than younger people because they have far less technological experience, and the physical and cognitive deterioration commonly associated with the older generation only exacerbates this issue [17,18]. However, there has also been a trend towards digital healthcare [19], which has many expected potential benefits for the elderly population specifically. Reliance on health technology systems would enable them to avoid travelling to a healthcare provider, should this be a challenge, or prolong the amount of time they can live at home rather than a nursing home [20]. Even personalization of services can provide potential through the system to reduce costs and achieve better overall outcomes [21].

There are numerous studies on the acceptability of new technologies for older people in the field of health. One study conducted [22] assessed the factors that facilitate the use and acceptance of wearable sensors in older people to monitor their health. It concluded that perceived usefulness, compatibility, ease of use, and the possibility of user feedback in the form of a health status score are vital. Additional studies [23,24] used exergaming for people over 65, focusing on summarising how researchers come to know the acceptability of the technology. It concluded that the following aspects are important: acceptability, efficiency/effectiveness/benefits, satisfaction, relevance (appropriateness), fun/enjoyment, challenges, feasibility, safety, interaction, usability, usefulness, and ease of use. Also worth mentioning is a work [25] in which the authors evaluated the importance of the family environment in the implementation of technological measures for older people. The results show that involving all family members in helping the person with the technological tool is significantly satisfying for them.

Based on the analysed literature, progress has been made to adapt the system to meet the needs of people diagnosed with different diseases, which is a good first step in personalisation. However, we must take into consideration the different ways of interacting with technology depending on other characteristics, such as country of origin, gender, age, or comfortability with technology. A study [26] based on the use of technology among the elderly found that while 78.3% of the 65–85 year olds they sampled in Cyprus used cell phones, there was a stark gap between genders. About 93.3% of men relied on mobile phones compared to only 59.4% of women. Therefore, it is possible that older women have different needs in their digital health applications than older men. Additionally, older individuals with higher income and levels of education have demonstrated a more substantial ability to adapt to and use new technologies [17]. A cross-sectional study [27] looked at the perspective of people with Parkinson’s, and they found that 28% of the participants reported unmet needs in relation to their disease. These studies also showed that those over 65 believed technology could help them with health problems and understanding care, those over 75 were less likely to use electronic methods for their healthcare. Thus, further customisation is necessary to ensure the system is as helpful as possible to its users.

Adapting technology within the healthcare domain opens up new possibilities and functionalities, such as the integration of recommendation systems, which have the potential to enhance personalized healthcare delivery and decision-making processes. An example was developed in a trial study [28], where patients achieved an 88% independency in managing medications thanks to personalised recommendations. In the TeNDER system, work on interfaces personalization included the development of the recommendation system module [29], which was piloted for 4–6 weeks, involving patients, carers and professionals. At the end of the pilot, and after analysing the surveys provided, it was determined to be perceived as user-friendly and having impacted positively the quality of life of the involved patients. Apart from recommendation systems, another study [30] proposed a framework for health-related searches, considering the patient as the highest weighting criteria.

Thanks to the TeNDER system, patients and their carers can monitor their state of health and receive feedback on the rehabilitation exercises carried out at the therapy centre. The system provides assistance by using the recommendation system, which uses the information obtained from the sensors and the interaction with other functionalities of the mobile app. It sends users advice, warnings, and recommendations for areas of their daily life such as physical activity, social aspects, nutrition, or sleep. Likewise, professionals obtain objective data on the state of health of patients that they can visualise thanks to the web app, enabling closer monitoring. Professionals can contact patients and caregivers through the communication tools of the TeNDER system, for example, by including appointments or reminders in the calendar of the patient/caregiver app.

The aim of this article is to show the developments throughout the TeNDER Project in the field of personalisation of interactions, how end-users collaborated in the co-design, and how, through a dynamic and continuous process, their needs have been incorporated.

## 2. Materials and Methods

The study detailed below was developed within the framework of the H2020 TeNDER project and lasted 42 months. The process involved end-users from 4 different countries (Spain, Italy, Germany, and Slovenia) with 3 different profiles (patients, carers, and health and social care professionals). All patients included were over 60 years of age, with a mean age of 74.5. In addition, the average age of the caregivers was 60 years and the average age of the professionals was 40 years. Throughout the process, around 1500 people were included, of whom 695 were patients, 469 carers, and 314 professionals.

Given that the study had different phases, the participation of the subjects was heterogeneous. The study was divided into two phases, the first of which included the collection and validation of initial user requirements and the second of which was subdivided into 3 consecutive waves of pilots, in which users were able to experience the developments implemented according to the needs and specifications collected. During the pilot, users could make use of different sensors and devices (smartwatch, binary and environmental sensors, deep cameras, sleep sensors, speakers, microphones, among others) and test TeNDER App version 0.9.9 on Android, which included feedback from the devices, communication services with professionals and carers, and recommendation system as main functionalities.

At the beginning of the users’ participation, they were asked about their technological affinity, and it could be observed that in the case of professionals, this affinity was almost 100%; in the case of carers, it was around 70%; and in the case of patients, it was limited to 60%. It is observed that technological affinity is inversely related to age: the older the age, the lower the degree of technological affinity reported. A certain bias in this respect can also be reported in relation to the acceptance of participation in a trial that involved use of technology.

### 2.1. Co-Design Process

The TeNDER system was developed in the framework of the TeNDER Project. The project lasted 42 months, during which time, to ensure the acceptability and usability of the TeNDER system, a careful co-design process was carried out during three different waves (patient distribution by wave in Table 1) to guarantee a person-centred approach. The process was present in all phases of system development and involved a continuous evolution of gathering direct feedback from end-users and implementing their needs and requirements.

The co-design process began with a literature review to establish the state of the art, after which the researchers involved in the project defined a methodology for collecting user requirements, implementing and validating them. The process was divided into several phases:

1. Collection of user requirements through a survey in 4 countries (Spain, Italy, Slovenia, and Germany) for each of the end-user types (patients, carers, and professionals) with the participation of 100 patients, 100 carers, and 50 health and social care professionals. In addition, 15 face-to-face interviews with patients, 15 with carers, and 15 with professionals were carried out. With the information collected, the first version of the TeNDER system was developed.
2. The first wave of pilots was carried out between November 2020 and April 2021 and involved 124 patients, 73 carers, and 53 professionals. During this wave, health and social care professionals, carers, and patients were able to test the first version of the system in real settings (home, hospital, day care centre, and rehabilitation ward) for periods ranging from 2 to 10 weeks. After the pilot test, data related to the usability, usefulness, and satisfaction as well as all kinds of qualitative comments with proposals for improvement and modification of the system were collected. With the results obtained in the first wave of pilots, the functionalities that were perceived as necessary and useful by the different end-users were prioritised, and improvements were made according to the needs described by the participants.
3. The second wave of testing took place between November 2021 and June 2022 and involved 250 patients, 104 carers, and 38 professionals. The co-design process at this stage was a cyclical procedure of collecting feedback and improving the system, leading to a final version of the system. After the second wave, improvements were aimed at polishing the interfaces and the presentation of information in both the web app and the mobile app.
4. The last wave was mostly focused on deploying all the services into the system and allowing the patients and caregivers to experiment with the system. After this period, surveys and interviews were conducted, and the evaluation results are presented in detail in following sections.

The entire process was important to develop the system and to adapt it to the needs of the different patients. There were some findings during the co-design process, which are summarized in Table 2. An important aspect of this digital health system for patients is its ability to empower them in the knowledge and management of their own health. The application provides assistance in adherence to therapies and treatments, as well as information that aids in decision-making processes related to patients’ health. Caregivers benefit from the system in that they can learn more about their loved ones’ health status, interact with professionals, and receive alert notifications when patients are in worrying or dangerous situations.

The customisation of the system should take into account these different objectives, as well as the different technological preferences of end-users. Patients and caregivers indicated that a mobile or tablet application is more accessible. Meanwhile, professionals would prefer not to rely on a mobile app, as smaller screens make it more difficult to easily view large amounts of patient lists and data at once. Professionals need to have access to patient lists and data, while caregivers and patients themselves should not have this function on their screens. In addition, the system must be able to adapt to the progression of the disease, as patients’ needs are likely to change. Another aspect of the system for which customisation was recommended was the frequency of reporting. Both patients and carers agreed that the structure of alerts and reminders should be modified. Carers felt that they should receive priority notifications about urgent alerts and receive information if a patient has not completed an activity that they had been reminded about. Patients also expressed a desire for more vital reminders to be distinguished from less urgent ones.

Further important factors in customising the system for each user are the specific characteristics that would influence the user experience. These include age, gender, education level, technological affinity, country of origin, primary disease, and associated comorbidities. It is also important to consider aspects such as marital status, lifestyle habits such as smoking, alcohol consumption, physical activity, sleep quality, rural or urban living environment, and whether living alone or in company.

Another frequent suggestion from end-users was the possibility of choosing the display modes and functionalities to be used. An added form of personalisation, according to the profiles of patients, carers, and professionals, would be to create open options for functionalities and display modes.

Finally, all three user groups recommended that the system should include real-time activity tracking, more precise functions to enable better understanding of reports, and a multi-modal tool for alerts to make the system more accessible.

### 2.2. TeNDER Integrated Components

The importance of personalization cannot be overstated, particularly when catering to the diverse needs of ageing populations. By considering factors such as country of origin, gender, age, and comfort with technology, we aim to create health technology systems that truly address the requirements of individual users. Various modules were developed within the project, which greatly benefited from the personalised interfaces developed through a patient-centric approach.

A pivotal aspect in the implementation of these modules has been the co-design process, wherein end-users actively participate in the development and refinement of the interfaces. This patient-centric approach recognizes that without involving patients and their perspectives in the design process, the resulting system would not genuinely align with their needs or preferences. By doing so, we not only acknowledge the significance of the ageing population and the challenges faced by healthcare systems but also acknowledge the potential benefits that new technologies can bring in addressing these challenges. The following presents the components developed within the project, highlighting how each module leverages the principles of personalization and co-design.

- An Electronic Health Record (EHR) acts as the TeNDER system backend, which was developed using HL7 standards [30]. HL7 is a widely used communication protocol technology that allows for the exchange of clinical data, allowing internal researchers to manage data from patients and observations from the sensorial devices. The co-design effort not only facilitated easy access to patient information for healthcare providers but also contributed to error reduction and enhanced patient safety.
- The multimodal fusion (MMF) module helps to provide a more complete view of patient data, enabling the system to provide more individualised recommendations to patients and helping both professionals and caregivers to better understand patients’ health status. In addition, the MMF module tracks the emotional state and irregular behaviour of patients, sending important notifications and recommendations to both caregivers and patients.
- The virtual assistant module consists of two sub-modules, the reminder sub-module and the chat sub-module, with the aim of facilitating patient interaction with the system. It incorporates voice commands for the components installed in users’ homes so that interaction with the system is carried out via voice command. By incorporating speech analytics, events such as time inquiries or caregiver notification requests were detected, enhancing user experience and accessibility.
- User profiling, a co-design-driven feature, combines sensory ecosystem data with patient information to establish connections between health status and daily behaviour patterns. User profiling aims to group patients according to common characteristics, which helps to personalise recommendations for each patient group. To perform user profiling, this component uses personal information such as gender, age, country, language, diagnoses among the three main TeNDER diseases, comorbidities, and multimodal fusion data.
- Finally, the recommendation system (RS) receives the results of the user profile and the information retrieved through the personalised patient questionnaires. The system uses this input information to detect triggering situations that require recommendations to improve patients’ daily lives. Observations from the devices worn by patients are converted into static data, which in turn trigger the messages of the recommendation tool. Most of the recommendation areas were covered by the co-design process analysing the daily aspects that needed to be addressed to improve the quality of life of patients.

The integration of these five components within the TeNDER system empowers users to gain a comprehensive understanding of their condition and daily health data. With access to real-time measurements and interactions through the mobile app, users receive personalized alerts, notifications, and recommendations based on recorded parameters. The system’s simplicity and personalization enable effortless interaction, allowing users to easily communicate with their caregivers and healthcare professionals, ensuring timely assistance when needed. These functionalities collectively contribute to enhancing users’ quality of life, preserving their autonomy, and providing accurate information for informed health decisions, ultimately fostering user empowerment.

### 2.3. Evaluation Methods

To comprehensively evaluate the TeNDER system, a pilot test was conducted in the 3rd wave. This evaluation aimed to have patients use the overall development of the system and evaluate its customised functionalities tailored specifically for them.

Patients and caregivers actively interacted with the health technology system through a mobile application, which they accessed on a daily basis. The app allowed them to monitor their health data and receive personalised recommendations and alerts. In addition, depending on the setting in which they participated (with settings including rehabilitation wards, hospitals, day care centres, and their own homes), users made use of different devices integrated into the system, such as cameras, sensors, wristbands, and others, providing real-life data that were incorporated into the system and allowed them to personalise interactions, recommendations, and alerts.

Following the experience, participants were invited to participate in a post-intervention interview. The interview was conducted face-to-face in most cases, and a telephone format was used for those who were unable to travel. The interview included the System Usability Scale (SUS) [31] questionnaire; a short set of questions about the degree of satisfaction, called Rate of Satisfaction (RS), with Likert-scale answers; and finally, a set of open-ended questions in response to which participants were invited to express in their own words how they felt using the system, what aspects they valued most positively, and what they liked least about the system, as well as what improvements they would incorporate to make it more attractive. The surveys were designed from the point of view of the end-user of the system (not from the technical side), taking into account that patients are often unfamiliar with the technology. The questions are detailed below:

1. SUS: [27] The user evaluates on a Likert-type scale with 5 response options whether he/she agrees or disagrees with the following statements.
  o SUS1: I think that I would like to use this system frequently.
  o SUS2: I found the system unnecessarily complex.
  o SUS3: I thought the system was easy to use.
  o SUS4: I think that I would need technical support.
  o SUS5: I found the various functions in this system were well integrated.
  o SUS6: I thought there was too much inconsistency in this system.
  o SUS7: I would imagine that most people would learn to use this system very quickly.
  o SUS8: I found the system very cumbersome to use.
  o SUS9: I felt very confident using the system.
  o SUS10: I needed to learn a lot of things before I could get going with this system.
2. RS: The user evaluates on a Likert-type scale with 5 response options whether he/she agrees or disagrees with the following statements. In the case of professionals, only the first two satisfaction questions were included.
  o RS1: How satisfied are you with the TeNDER system?
  o RS2: Rate your experience with the TeNDER system.
  o RS3: How satisfied are you with reports about your activities and progress?
  o RS4: How satisfied are you with the overview of your health status and events from TeNDER?
3. Open questions to collect direct feedback: The user responds in their own words describing their experience.
  o OQ1: How do you feel about the TeNDER system?
  o OQ2: What do you like less about the TeNDER system?
  o OQ3: What do you like more about the TeNDER system?
  o OQ4: What would you change in order to make the TeNDER System more useful and applicable for you?

## 3. Results

This pilot involved a wide range of end-users, including patients, carers, and professionals (more detailed information in Table 3) from four countries (Spain, Germany, Italy, and Slovenia). The test lasted between 2 and 8 weeks, during which users had the opportunity to interact with the system in a variety of settings, including rehabilitation wards, hospitals, day centres, and their own homes.

The results section of this study presents the findings obtained through the evaluation of the TeNDER system. The SUS and the RS were the two primary instruments used to measure usability and satisfaction with the system. All the results presented in this section include the data from the surveys completed by the patients, caregivers, and professionals.

First, Table 4 and Table 5 display the SUS scores of 167 patients who completed all the surveys.

Over 65% of patients participating in the pilot said they would use the system frequently, and over 41% said the system was easy to use, based on information extracted from Table 4.

More than 70% of the patients participating in the pilot reported being satisfied or very satisfied with the system, and almost 41% of them said they were satisfied with the reports the system provides them (Table 5). Out of the total 167 caregivers, 129 of them were caregivers providing valuable information who completed all the surveys. Results for caregivers are presented in Table 6 and Table 7.

Almost 70% of caregivers felt confident using the system, as extracted from the System Usability Scale on Table 6.

More than 65% of carers are satisfied with the system (Table 7). Finally, the results from the surveys for the professionals are presented in Table 8 and Table 9. In this case, 77 participants filled in all the surveys out of the total number (95).

More than 75% of practitioners reported that the various components of the system were well integrated.

The percentage of professionals who say they are satisfied with the system is around 60%.

Finally, in relation to the open questions offered by the pilot participants, it is worth noting the feelings of reassurance that were reflected regarding the use of the system and the receipt of the personalised alerts and recommendations. Likewise, the usefulness of the personalised recommendations was underlined, specifying that they have helped participants to lead healthy lifestyles and that they have been easy to implement on a day-to-day basis. Some users indicated that it helped them to implement routines in their daily lives that they already knew were useful but had forgotten to incorporate. There is a general perception that it is more positive not to receive many recommendations, but that these should be well adapted and specific.

## 4. Discussion

The TeNDER System was created with the aim of creating a space where patients, carers, and professionals can have access to objective information in real time on the state of patients. The system also offers notifications, warnings, and recommendations based on the information collected by sensors, devices, and interaction with the app, aiming to have a positive impact on perceived quality of life and autonomy, as well as on informed health decision making.

The study was conducted in four different countries (Spain, Italy, Germany, and Slovenia) and included three different chronic diseases (Parkinson’s, Alzheimer’s, and cardiovascular pathology). People from different socio-cultural backgrounds were involved, although it is true that the need to make use of technology may have led to a certain bias in the inclusion of users. The study provided all the devices to the participants, including the smartphones used in the study. The system was developed in English and adapted to the languages of the four pilot countries. This can be considered the main difference from the related work [30] mentioned in the literature, where they were not able to extract a proper analysis, as there are not many tests created at a large scale, due to there not being many cases vs. control studies. TeNDER addressed that by conducting a study in many different countries.

Thanks to the results tables reported in the previous section, we can see that all end-users, patients, carers, and professionals, had mostly positive opinions about the usability and usefulness of the system. It also highlights a high percentage of acceptability, taking as a reference the first question of the SUS questionnaire, where it is stated that they were willing to use the system frequently.

Looking at the answers to the open-ended questions, we found that 70% of patients participating in the pilot reported positive feelings about using the system, such as feeling safe, reassured, motivated, satisfied, or relaxed. Some reported feeling in control all the time, and others reported feeling insecure or nervous due to their lack of technological knowledge. Similar responses appear in the case of the caregivers, of whom 60% report feeling good and more confident thanks to the use of the application. Some carers said that they did not make much use of the app, and a few said that they found it difficult to use the app due to their lack of knowledge, similar to what happened in the case of patients.

Two main conclusions can be drawn from the professionals’ responses: 75% of the professionals involved say that the purpose of the system is useful and can support them in their daily work; however, they state that there is room for improvement and that it would be good to include more detailed reports.

Among the aspects that were least liked were the short battery life of the bracelets, as well as some connectivity failures and the fact that the system is limited to specific pathologies. As a highlight, it is worth mentioning the perceived usefulness of the data collected and displayed by the system and the motivation that this monitoring provides for many of the participants.

Finally, participants were asked what aspects they would improve to make the system more useful and attractive. First among these was the resolution of errors or possible technical problems that arose in some cases. Terms such as simplicity, intuitiveness, and accessibility were also mentioned in numerous responses.

On the other hand, the incorporation of new functionalities, the extension of the recommendations, and the inclusion of more questionnaires that provide additional information are elements of responses that are repeated.

## 5. Conclusions

The inclusion of technological systems in the field of health is an area of great interest at the present time. It allows for a more efficient approach and reduces the economic burden on health systems. The population pyramid featuring a growing number of elderly individuals poses a great challenge for the sustainability of welfare systems in European countries, and the inclusion of new technologies and care models such as the one proposed by the TeNDER system appears to be really attractive based on results.

However, we must bear in mind the limitations of access, usability, and acceptability that these systems may have in the elderly population, even more so in people with chronic pathologies. It has been shown that those systems built under a person-centred approach, involving the end-users in the design of the solutions from the very beginning, are more accepted and more useful than those in which this approach is not developed.

The TeNDER System has undergone a careful co-design process that has resulted, among other actions, in a greater customisation of interfaces, user interactions with the system, and the system’s recommendations and suggestions. All this has helped to increase the degree of user satisfaction with the system. Even so, there is still a long way to go, and actions must continue to be taken to bring technical developers closer to the real and particular needs of end-users.

Future research could explore how to effectively integrate digital healthcare into existing healthcare systems and policies, to ensure that these systems can be widely adopted and used by older adults.

The work carried out in this study is aligned with the UN Sustainable Development Goal 3: Health and Well-being.

## Figures and Tables

**Table 1:** Patient distribution by main disease along project lifetime.

Main Disease	1st Wave	2nd Wave	3rd Wave
PD	43 (34.6%)	62 (24.8%)	112 (34.9%)
AD	45 (36.2%)	99 (39.6%)	86 (26.8%)
CVD	36 (29.0%)	89 (35.6%)	123 (38.3%)
Total	124 (100%)	250 (100%)	321 (100%)
Mean age	72.7 (±8.6)	73.9 (±10.3)	74.4 (±7.8)

**Table 2:** Co-design interventions for system personalization.

Recommendation	Why?	Target User
Simplicity of interfaces/functionality	The system has to be easy to use for patients that are not digitally active	All, especially “patients”. Only 18% are familiar with technology
Interface fonts	Patients might have problems reading/finding a functionality	Patients, carers
Interface functions	Simple screens, with few components per layout	Patients, carers
Customisation	Not all the functionalities are useful to all the users	Patients, carers, some professionals
Data Access	Not all the roles want/can access patients’ data	Professionals
Frequency of reports	Customisation might be enabled	All users
Structure of the alerts	Carers shall have priority view/notification of urgent alerts	Carers
Structure of the reminders	To distinguish more important/vital reminders from others	Patients
Feedback from the reminders	Carers shall have feedback if the action has (not) been taken by a patient	Patients, carers
Performance to be shown	To encourage and increase motivation	Patients
Real-time activity tracking	Enabling the proper performance of exercises, assuring safety and security	All
Accuracy of the functions	To avoid wrong impressions and misinterpretation of the reports	All
Multimodal tool for the alerts (voice, text message, e-mail)	To allow broader usage according to the need and technology acceptance	All
Modularity of the system	The progression of the diseases may cause different needs	Patients, carers
Affordability	The system shall be designed in a way the community can afford and benefit from	All

**Table 3:** General information from 3rd wave about patients, caregivers, and professionals.

General	Type	Total	Case	Control	*p*-Value	Caregivers	Professionals
Country					0.05		
	Spain	165 (51.4%)	105 (63.6%)	60 (36.4%)		53 (31.7%)	49 (51.6%)
	Italy	44 (13.7%)	20 (45.5%)	24 (54.5%)		44 (26.3%)	26 (27.4%)
	Germany	66 (20.6%)	34 (51.5%)	32 (48.5%)		31 (18.5%)	15 (15.8%)
	Slovenia	46 (14.3%)	31 (67.4%)	15 (32.6%)		39 (23.3%)	5 (5.2%)
Gender					0.66		
	Female	147(45.8%)	85 (57.8%)	62 (42.2%)		108 (64.7%)	75 (79.0%)
	Male	173 (54.2%)	104 (60.1%)	69 (39.9%)		59 (35.3%)	20 (21.0%)
Main disease					0.51		
	AD	112 (34.9%)	71 (63.4%)	41 (36.6%)		93 (55.6%)	N/A
	PD	86 (26.8%)	48 (55.8%)	38 (44.2%)		52 (31.1%)	N/A
	CVD	123 (38.3%)	71 (57.7%)	52 (42.3%)		22 (13.1%)	N/A
Scenario					0.04		
	Home	161 (50.2%)	105 (65.2%)	56 (34.8%)		70 (41.9%)	N/A
	Day care centre	13 (4.0%)	10 (76.9%)	3 (23.1%)		10 (5.9%)	N/A
	Hospital	109 (34.0%)	54 (49.5%)	55 (50.5%)		68 (40.7%)	N/A
	Rehabilitation room	38 (11.8%)	21 (55.3%)	17 (44.7%)		19 (11.3%)	N/A

**Table 4:** System Usability Scale results table for patients.

Questionnaire	Strongly Disagree	Disagree	Nor Agree or Disagree	Agree	Strongly Agree
SUS1	20 (10.2%)	20 (11.9%)	20 (12.0%)	56 (33.5%)	54 (32.3%)
SUS2	47 (28.1%)	60 (35.9%)	34 (20.4%)	17 (10.2%)	9 (5.4%)
SUS3	8 (4.8%)	20 (11.9%)	29 (17.4%)	62 (37.1%)	48 (4.8%)
SUS4	15 (9.0%)	1 (0.6%)	21 (12.6%)	42 (25.2%)	59 (35.3%)
SUS5	9 (5.4%)	11 (6.5%)	65 (38.9%)	48 (28.7%)	34 (20.4%)
SUS6	23 (13.8%)	49 (29.3%)	78 (46.7%)	7 (4.2%)	10 (5.9%)
SUS7	5 (3.0%)	24 (14.3%)	40 (23.9%)	50 (29.9%)	48 (28.7%)
SUS8	40 (24.0%)	64 (38.3%)	28 (16.8%)	24 (14.4%)	11 (6.6%)
SUS9	11 (6.6%)	19 (11.4%)	26 (15.6%)	38 (22.8%)	73 (43.7%)
SUS10	40 (24.0%)	32 (19.2%)	30 (18.0%)	40 (23.9%)	25 (14.9%)

**Table 5:** Rate of Satisfaction results table for patients.

Questionnaire	Very Unsatisfied	Unsatisfied	Neutral	Satisfied	Very Satisfied
RS1	11 (6.6%)	6 (3.6%)	32 (19.2%)	87 (52.1%)	31 (18.6%)
RS2	3 (1.8%)	1 (0.6%)	59 (35.3%)	3 (1.8%)	101 (60.5)
RS3	16 (9.6%)	17 (10.2)	59 (35.3%)	75 (44.9%)	0 (0.0%)
RS4	1 (0.6%)	16 (9.6%)	45 (26.9%)	73 (43.7%)	32 (19.2%)

**Table 6:** System Usability Scale results table for caregivers.

Questionnaire	Strongly Disagree	Disagree	Nor Agree or Disagree	Agree	Strongly Agree
SUS1	3 (2.3%)	12 (9.3%)	21 (16.3%)	53 (41.1%)	40 (31.0%)
SUS2	25 (19.4%)	58 (44.9%)	31 (24.0%)	13 (10.1%)	2 (1.6%)
SUS3	1 (0.8%)	16 (12.4%)	19 (14.7%)	68 (52.7%)	25 (19.4%)
SUS4	14 (10.9%)	28 (21.7%)	26 (20.2%)	44 (34.1%)	17 (13.2%)
SUS5	4 (3.1%)	6 (4.7%)	49 (37.9%)	62 (48.1%)	8 (6.2%)
SUS6	23 (17.8%)	48 (37.2%)	51 (39.5%)	6 (4.7%)	1 (0.8%)
SUS7	3 (2.3%)	20 (15.5%)	36 (27.9%)	46 (35.7%)	24 (18.6%)
SUS8	31 (24.0%)	55 (42.6%)	20 (15.5%)	18 (13.9%)	5 (3.9%)
SUS9	1 (0.8%)	11 (8.5%)	27 (20.9%)	52 (40.3%)	38 (29.5%)
SUS10	31 (24.0%)	45 (34.9%)	26 (20.2%)	23 (17.8%)	4 (3.1%)

**Table 7:** Rate of Satisfaction results table for caregivers.

Questionnaire	Very Unsatisfied	Unsatisfied	Neutral	Satisfied	Very Satisfied
RS1	3 (2.3%)	10 (7.8%)	31 (24.0%)	68 (52.7%)	17 (13.2%)
RS2	3 (2.3%)	5 (3.9%)	52 (40.3%)	0 (0.0%)	69 (53.5%)
RS3	1 (0.8%)	44 (34.1%)	49 (37.9%)	44 (34.1%)	22 (17.0%)
RS4	1 (0.8%)	11 (8.5%)	47 (36.4%)	50 (38.8%)	20 (15.5%)

**Table 8:** System Usability Scale results table for professionals.

Questionnaire	Strongly Disagree	Disagree	Nor Agree or Disagree	Agree	Strongly Agree
SUS1	0 (0.0%)	0 (0.0%)	16 (20.8%)	42 (54.6%)	19 (24.7%)
SUS2	24 (31.2%)	24 (31.2%)	24 (31.2%)	4 (1.3%)	1 (1.3%)
SUS3	1 (1.3%)	3 (3.9%)	17 (22.0%)	39 (50.7%)	17 (22.1%)
SUS4	34 (44.2%)	24 (31.2%)	6 (7.8%)	13 (16.9%)	0 (0.0%)
SUS5	0 (0.0%)	1 (1.3%)	16 (20.8%)	51 (66.2%)	9 (11.7%)
SUS6	22 (28.6%)	28 (36.4%)	25 (32.5%)	2 (2.6%)	0 (0.0%)
SUS7	0 (0.0%)	4 (5.2%)	21 (27.3%)	36 (46.8%)	16 (20.8%)
SUS8	25 (32.5%)	38 (49.4%)	11 (14.3%)	3 (3.9%)	0 (0.0%)
SUS9	0 (0.0%)	1 (1.3%)	29 (37.7%)	33 (42.9%)	14 (18.2%)
SUS10	25 (32.5%)	29 (37.7%)	13 (16.9%)	9 (11.7%)	1 (1.3%)

**Table 9:** Rate of Satisfaction results table for professionals.

Questionnaire	Very Unsatisfied	Unsatisfied	Neutral	Satisfied	Very Satisfied
RS1	0 (0.0%)	3 (3.9%)	28 (36.4%)	44 (57.1%)	2 (2.6%)
RS2	0 (0.0%)	3 (3.9%)	40 (51.9%)	1 (1.3%)	33 (42.9%)

## Data Availability

Not applicable.

## References

[1] (2021). Global Strategy on Digital Health 2020–2025. http://apps.who.int/bookorders.

[2] National Digital Health Strategy and Framework for Action|Australian Digital Health Agency. https://www.digitalhealth.gov.au/about-us/strategies-and-plans/national-digital-health-strategy-and-framework-for-action.

[3] New Zealand Health Strategy: Future Direction|Ministry of Health NZ. https://www.health.govt.nz/new-zealand-health-system/new-zealand-health-strategy-future-direction.

[4] Digital Health and Care—GOV.UK. https://www.gov.uk/government/publications/uk-life-sciences-support/digital-health-and-care.

[5] Home|España Digital 2026. https://espanadigital.gob.es/.

[6] Sistema Nacional de Salud (2021). Estrategia de Salud Digital. https://www.sanidad.gob.es/ciudadanos/pdf/Estrategia_de_Salud_Digital_del_SNS.pdf.

[7] (2020). eHealth Strategy for Ireland. https://www.gov.ie/en/publication/6b7909-ehealth-strategy-for-ireland/.

[8] SmartHealthSystems. https://www.bertelsmann-stiftung.de/en/publications/publication/did/smarthealthsystems-1.

[9] Solachidis V., Moreno J.R., Hernández-Penaloza G., Vretos N., Álvarez F., Daras P. TeNDER: Towards efficient Health Systems through e-Health platforms employing multimodal monitoring. Proceedings of the 2021 IEEE/ACM Conference on Connected Health: Applications, Systems and Engineering Technologies (CHASE).

[10] Belmonte-Hernández A., Theodoridis T., Burgos González M., Hernández-Peñaloza G., Solachidis V., Jiménez Ramos J., Álvarez F. A Novel Framework for Physical Therapy Rehabilitation Monitoring and Assessment in Parkinson Disease Patients using Depth Information. Proceedings of the PETRA ′19: The 12th ACM International Conference on PErvasive Technologies Related to Assistive Environments.

[11] Talukder M.S., Sorwar G., Bao Y., Ahmed J.U., Palash M.A.S. (2020). Predicting antecedents of wearable healthcare technology acceptance by elderly: A combined SEM-Neural Network approach. Technol. Forecast. Soc. Chang..

[12] Avgerinos C., Solachidis V., Vretos N., Daras P. (2020). Non-Parametric Clustering Using Deep Neural Networks. IEEE Access.

[13] Jiménez J., Mercado J., Carrasco L., Ruíz V., Solachidis V., Serrano J. Monitoring of motor function in the rehabilitation room. Proceedings of the PETRA ′22: The 15th International Conference on PErvasive Technologies Related to Assistive Environments.

[14] Corselli-Nordblad L., Strandell H. (2020). Ageing Europe: Looking at the Lives of Older People in the EU: 2020 Edition.

[15] Harley D.A., Kurniawan S.H., Fitzpatrick G., Vetere F. Age matters: Bridging the generation gap through technology-mediated interaction. Proceedings of the CHI EA ′09: CHI ′09 Extended Abstracts on Human Factors in Computing Systems.

[16] Aggarwal M., Rawat M.S., Singh S., Srivastava S., Gauba P. (2017). Generation Gap: An Emerging Issue of Society. Int. J. Eng. Technol. Sci. Res..

[17] Fischer S.H., David D., Crotty B.H., Dierks M., Safran C. (2014). Acceptance and use of health information technology by community-dwelling elders. Int. J. Med. Inform..

[18] Mostaghel R. (2016). Innovation and technology for the elderly: Systematic literature review. J. Bus. Res..

[19] Cumming G.P., McKendrick D., Hogg J., French T., Kahana E., Molik D., Luciano J.S. (2017). Formulating ehealth utilizing an ecological understanding. Digit. Healthc. N. Chall. Oppor..

[20] Deen M.J. (2015). Information and communications technologies for elderly ubiquitous healthcare in a smart home. Pers. Ubiquitous Comput..

[21] Cancela J., Charlafti I., Colloud S., Wu C. (2021). Digital health in the era of personalized healthcare: Opportunities and challenges for bringing research and patient care to a new level. Digit. Health Mob. Wearable Devices Particip. Health Appl..

[22] Li J., Ma Q., Chan A.H., Man S. (2019). Health monitoring through wearable technologies for older adults: Smart wearables acceptance model. Appl. Ergon..

[23] Nawaz A., Skjæret N., Helbostad J.L., Vereijken B., Boulton E., Svanaes D. (2016). Usability and acceptability of balance exergames in older adults: A scoping review. Health Inform. J..

[24] Skjæret N., Nawaz A., Morat T., Schoene D., Helbostad J.L., Vereijken B. (2016). Exercise and rehabilitation delivered through exergames in older adults: An integrative review of technologies, safety and efficacy. Int. J. Med. Inform..

[25] Luijkx K., Peek S., Wouters E. (2015). Grandma, You Should Do It—It’s Cool’ Older Adults and the Role of Family Members in Their Acceptance of Technology. Int. J. Environ. Res. Public Health.

[26] The Use of Technology by the Elderly. https://www.researchgate.net/publication/266448717_The_use_of_technology_by_the_elderly.

[27] Duroseau N., Abramson T., Pergament K., Chan V., Govindavari J.P., Ciraco C. (2016). Acceptance of technology-based tools in a sample of Parkinson’s patients. Chronic Illn..

[28] Mira J.J., Navarro I., Botella F., Borrás F., Nuño-Solinís R., Orozco D. (2014). A Spanish pillbox app for elderly patients taking multiple medications: Randomized controlled trial. J. Med. Internet Res..

[29] del Rio A., Jimenez J., Medina-García R., Lozano-Hernández C., Alvarez F., Serrano J. (2023). Improving Quality of Life in Chronic Patients: A Pilot Study on the Effectiveness of a Health Recommender System and Its Usability. Appl. Sci..

[30] Cumming G.P., French T., Hogg J., McKendrick D., Gilstad H., Molik D., Luciano J.S. (2017). Trust and provenance in communication to ehealth consumers. Digit. Healthc. N. Chall. Oppor..

[31] Affairs A.S. System Usability Scale (SUS). https://www.usability.gov/how-to-and-tools/methods/system-usability-scale.html.

